# Complete mitochondrial genome and the phylogenetic position of the stellate puffer (*Arothron stellatus*)

**DOI:** 10.1080/23802359.2019.1673224

**Published:** 2019-10-04

**Authors:** Wei-Feng Chen, Xin Peng, Yue Yu, Can-Min Yang, Xiao Chen, Jun Zhao, Junjie Wang

**Affiliations:** a Zhejiang Mariculture Research Institute, Wenzhou, Zhejiang, P. R. China;; b School of Life Science, South China Normal University, Guangzhou, Guangdong, P. R. China

**Keywords:** *Arothron stellatus*, Tetraodontidae, Tetraodontiformes

## Abstract

The complete mitochondrial genome of the stellate puffer (*Arothron stellatus*) was first determined in this study. It was a 16,475 bp circular molecule and consisted of 37 genes with typical gene order in vertebrate mitogenome. Its nucleotide content was 29.0% A, 32.0% C, 15.9% G and 23.1% T. This mitogenome had 30 bp short intergenic spaces located in 12 gene junctions and 28 bp overlaps located in 7 gene junctions. In the protein-coding genes, two start codons (GTG and ATG) and two stop codons (TAG and TAA/TA/T) were found. The 22 tRNA genes ranged from 64 bp (tRNA-*Cys*) to 74 bp (tRNA-*Leu1* and tRNA-*Lys*). The phylogenetic result showed that *Takifugu xanthopterus* was most closely related to the reef-related *A. stellatus* among all Tetraodontiformes species.

The stellate puffer (*Arothron stellatus*) (Tetraodontidae, Tetraodontiformes), a reef-associated fish, is widespread in the coasts of Taiwan, the South China Sea, northward to southern Japan and southward to Lord Howe Island (Lieske and Myers 1994; Su and Li 2002). For most of them, growing to 54.0 cm is easy, however, only a few part of this population can reach the max length: 120 cm (Bouhlel 1988; Lieske and Myers 1994). The molecular and genetic research of this species was very little. In this study, we first determined the complete mitochondrial genome of *A. stellatus*, and analyzed the phylogenetic relationship of the fishes in Tetraodontiformes fishes.

Tissue of *A. stellatus* (voucher no. YS2017051325) was collected from Yongshu island, China. The experimental protocol and data analysis methods followed Chen et al. (2014). Excluding *A. stellatus* and the outgroup *Triacanthus biaculeatus* and *Trixiphichthys weberi*, 18 species from 10 families of Tetraodontiformes with the complete mitogenomes available in the Genbank were selected to construct the phylogenetic tree. The maximum-likelihood (ML) method was fulfilled with the AIC model in PhyML 3.0 by two partitions of the mitogenome: the first and second codons of the 12 protein-coding genes (except the light-strand encoded *ND6* gene) (Guindon et al. 2010).

The reef-associated *A. stellatus* mitochondrial genome was found to form a closed loop that is 16,475 bp long (Genbank accession number: MK391748). Its nucleotide base composition was 29.0% A, 32.0% C, 15.9% G, and 23.1% T. This circle molecular had a typical mitogenomic organization and gene order as most vertebrates, comprising 13 protein-coding genes, 22 tRNA genes, 2 rRNA genes, and an AT-rich control region. Whole mitogenome had 30 bp short intergenic spaces located in 12 gene junctions and 28 bp overlaps located in 7 gene junctions. The 13 protein-coding genes used two start codons (GTG and ATG) and two kinds of termination codons (TAG and TAA/TA/T) were identified, and most of them shared common initial codon ATG and terminal codon TAA. The 22 tRNA genes ranged from 64 bp (tRNA-*Cys*) to 74 bp (tRNA-*Leu1* and tRNA-*Lys*), among which six tRNA genes were located in the light strand, while the remaining tRNA genes were in the heavy strand. The 12S and 16S rRNA genes were located between the tRNA-*Phe* and tRNA-*Leu1* genes, separated by the tRNA-*Val* gene. A 35 bp inserted sequence was identified as the origin of light-strand replication (OL) between tRNA-*Asn* and tRNA-*Cys* genes with a stem-loop structure. The control region was 840 bp, presenting a high A + T content (60.6%).

The phylogenetic analysis suggested that *Takifugu xanthopterus* is more closely related to *A. stellatus* than it is to the other species concluded in the analysis (Figure 1). The taxonomic conclusion drew by phylogenetic tree was congruent with morphological taxonomy.

**Figure 1.**
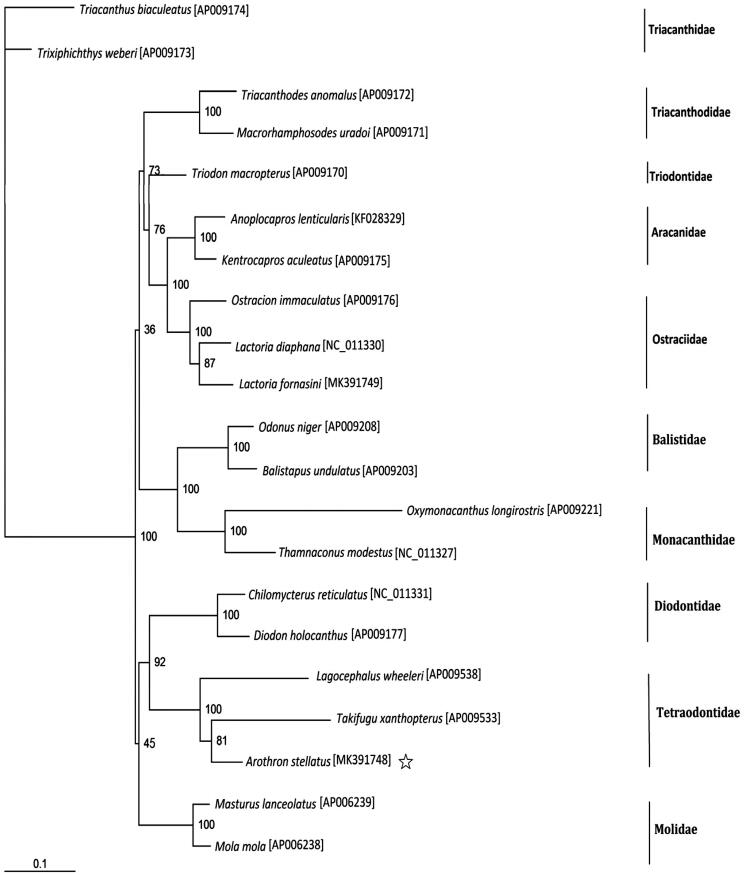
Phylogenetic position of *Arothron stellatus*. Phylogenetic relationships (maximum-likelihood) of species of the order Tetraodontiformes based on nucleotide sequence of 12 protein-coding genes in the mitochondrial genome. Numbers beside each node represent percentages of 100 bootstrap values.
